# Modification of erythrocyte membrane phospholipid composition in preterm newborns with retinopathy of prematurity: The omegaROP study

**DOI:** 10.3389/fcell.2022.921691

**Published:** 2022-09-09

**Authors:** Rémi Karadayi, Charlotte Pallot, Stéphanie Cabaret, Julie Mazzocco, Pierre-Henry Gabrielle, Denis S. Semama, Corinne Chantegret, Ninon Ternoy, Delphine Martin, Aurélie Donier, Stéphane Gregoire, Catherine P. Creuzot-Garcher, Alain M. Bron, Lionel Bretillon, Olivier Berdeaux, Niyazi Acar

**Affiliations:** ^1^ Centre des Sciences du Goût et de l’Alimentation, Institut Agro, CNRS, INRAE, Université Bourgogne Franche-Comté, Eye and Nutrition Research Group, Dijon, France; ^2^ University Hospital, Department of Ophthalmology, Dijon, France; ^3^ Centre des Sciences du Goût et de l’Alimentation, Institut Agro, CNRS, INRAE, Université Bourgogne Franche-Comté, ChemoSens Platform, Dijon, France; ^4^ University Hospital, Neonatal Intensive Care Unit, Dijon, France

**Keywords:** polyunsaturated (essential) fatty acids, plasmalogens, phospholipids, erythrocyte, retinopathy of prematurity

## Abstract

N-3 polyunsaturated fatty acids (PUFAs) may prevent retinal vascular abnormalities observed in oxygen-induced retinopathy, a model of retinopathy of prematurity (ROP). In the OmegaROP prospective cohort study, we showed that preterm infants who will develop ROP accumulate the n-6 PUFA arachidonic acid (ARA) at the expense of the n-3 PUFA docosahexaenoic acid (DHA) in erythrocytes with advancing gestational age (GA). As mice lacking plasmalogens ―That are specific phospholipids considered as reservoirs of n-6 and n-3 PUFAs― Display a ROP-like phenotype, the aim of this study was to determine whether plasmalogens are responsible for the changes observed in subjects from the OmegaROP study. Accordingly, preterm infants aged less than 29 weeks GA were recruited at birth in the Neonatal Intensive Care Unit of University Hospital Dijon, France. Blood was sampled very early after birth to avoid any nutritional influence on its lipid composition. The lipid composition of erythrocytes and the structure of phospholipids including plasmalogens were determined by global lipidomics using liquid chromatography coupled to high-resolution mass spectrometry (LC-HRMS). LC-HRMS data confirmed our previous observations by showing a negative association between the erythrocyte content in phospholipid esterified to n-6 PUFAs and GA in infants without ROP (rho = −0.485, *p* = 0.013 and rho = −0.477, *p* = 0.015 for ethanolamine and choline total phospholipids, respectively). Phosphatidylcholine (PtdCho) and phosphatidylethanolamine (PtdEtn) species with ARA, namely PtdCho16:0/20:4 (rho = −0.511, *p* < 0.01) and PtdEtn18:1/20:4 (rho = −0.479, *p* = 0.015), were the major contributors to the relationship observed. On the contrary, preterm infants developing ROP displayed negative association between PtdEtn species with n-3 PUFAs and GA (rho = −0.380, *p* = 0.034). They were also characterized by a positive association between GA and the ratio of ethanolamine plasmalogens (PlsEtn) with n-6 PUFA to PlsEtn with n-3 PUFAs (rho = 0.420, *p* = 0.029), as well as the ratio of PlsEtn with ARA to PlsEtn with DHA (rho = 0.843, *p* = 0.011). Altogether, these data confirm the potential accumulation of n-6 PUFAs with advancing GA in erythrocytes of infants developing ROP. These changes may be partly due to plasmalogens.

## Introduction

Retinopathy of prematurity (ROP) is the leading cause of childhood blindness with an estimated incidence ranging from 6 to 34% in developed countries (Good & Hardy, 2001; Jordan, 2014). ROP is characterized by a first phase of vaso-obliteration in the central retina (phase 1), followed by the overexpression of pro-angiogenic growth factors such angiopoietins and VEGF (Sonmez, Drenser, Capone, & Trese, 2008; Sato, Shima, & Kusaka, 2011; Rivera et al., 2017) associated to neovascular events in the retina (phase 2) (Hellstrom, Smith, & Dammann, 2013). As a result, mature retinal vessels exhibit several major abnormalities, such as increased dilatation and tortuosity, as well as vascular leakage (Hellstrom et al., 2013).

Previous studies have reported that the polyunsaturated fatty acids (PUFAs) such as arachidonic acid (ARA, C20:4 n-6) and docosahexaenoic acid (DHA, C22:6 n-3) influence retinal vascularization processes in mouse models of oxygen-induced retinopathy, a mouse model of ROP (Connor et al., 2007; Sapieha et al., 2011). Moreover, human studies revealed alterations in blood levels of PUFAs in preterm newborns developing ROP (Martin et al., 2011; Lofqvist et al., 2018; Pallot et al., 2019). Particularly in the OmegaROP study, we have shown an accumulation of ARA at the expense of DHA in erythrocytes of preterm infants that will develop ROP. In cell membranes, PUFAs such as ARA and DHA are esterified on membrane phospholipids, from which they can be released by phospholipases for further intracellular metabolization and/or signaling. Within these phospholipids, plasmalogens represent a particular sub-class characterized by the presence of a vinyl-ether bond at *sn*-1 position of glycerol instead of an ester bond. Plasmalogens are considered as “reservoirs” for PUFAs such as ARA and DHA (Nagan & Zoeller, 2001) and are abundant in the human retina (Bretillon et al., 2008; Berdeaux et al., 2010; Acar et al., 2012). Interestingly, we have shown that plasmalogen-deficient mice exhibit retinal vascular abnormalities resembling to those observed in ROP (Saab-Aoude, Bron, Creuzot-Garcher, Bretillon, & Acar, 2013; Saab et al., 2014). Indeed, retinal vascular development in these mice was characterized by a delayed outgrowth followed by increased angiogenesis associated to the overexpression of pro-angiogenic factors such as angiopoietins (Saab et al., 2014).

In this work, we aimed to assess whether the differential accumulation of ARA and DHA in preterm infants of the OmegaROP study is associated to plasmalogens. By using high-resolution mass spectrometry (HRMS), we evaluated the concentrations of individual phospholipids species in erythrocytes of preterm infants that will or not develop ROP. In this study, blood was collected immediately after birth, in order to limit the interference with lipid nutritional intakes.

## Materials and methods

### Ethics statement

This study was conducted in accordance with the guidelines of the Declaration of Helsinki. The experimental procedures were approved by local ethics committee (CPP Est III, School of Medicine, Dijon, France) that waived the obtainment of a written consent as our study did not generate additional procedure to those of standard care. Instead of, an information note was given to parents and/or legal guardians. In accordance with “ethical considerations for clinical trials on medicinal products conducted with the pediatric population”, the volume of blood collected in preterm infants was limited to 0.5 mL (European Union. 2008).

### Selection of the patients

As described previously (Pallot et al., 2019), all preterm infants born before 29 weeks GA and hospitalized in the neonatal intensive care unit of the Dijon University Hospital, Dijon, France, between 31 July 2015 and 31 January 2018 were included in the study. A 0.5-mL blood sample was collected by venipuncture in a heparinized tube within the first 48 h of life. Red blood cells were immediately separated from plasma by centrifugation at 1860 × g at 4°C. The red blood cell pellet was then washed three times with an isotonic saline solution. Samples were stored at −80°C until lipidomic analyses.

ROP screening was performed with the wide-field RETCAM II^®^ camera (Clarity Medical Systems; Pleasanton, CA, United States) using a lid speculum after topical anesthesia by chlorhydrate oxybuprocaine, 1.6 mg/0.4 mL (Théa, Clermont-Ferrand, France). Pupillary dilation was previously performed using one drop of tropicamide, 2 mg/0.4 mL, Théa, Clermont-Ferrand, France). The procedure was completed by a trained nurse and all fundus photographs were analyzed by two trained pediatrics-specialized ophthalmologists. Screening began at 4–6 weeks of life but never before 31 weeks of postconceptional age (PCA). Fundus imaging was repeated every other week until 39 weeks PCA if no ROP was detected, and every week or up to twice a week in case of ROP. ROP staging was determined according to the International Classification of ROP (International Committee for the Classification of Retinopathy of, 2005). Subjects were classified in the group suffering from ROP (ROP group) or in the group of unaffected controls (no-ROP group). Within the ROP group, subjects were classified into type 1 ROP and type 2 ROP. The major risks of developing ROP, namely gestational age (GA), weight at birth, duration of mechanical ventilation, sepsis, use of erythropoietin, red blood cell transfusion and cerebral hemorrhage were documented.

### Characterization and quantification of individual phospholipid species

Total lipids were extracted from red blood cells according to Moilanen and Nikkari by using chloroform/methanol (1:1, v:v) (Moilanen & Nikkari, 1981). The phosphorus content of the total lipid extracts was determined according to the method developed by Bartlett and Lewis (Bartlett & Lewis, 1970). The samples were then diluted at a concentration of 500 μg/mL in chloroform/methanol 1:1 (v/v). Quality control (QC) were prepared by pooling 10 µL of each resuspended lipid extract. The concentrations of individual phospholipids species of erythrocytes total lipids were determined by Hydrophilic Interaction Liquid Chromatography coupled to High Resolution Mass Spectrometry (HILIC-HR-MS).

Liquid chromatography analyses were performed using a Dionex UltiMate™ 3000 LC pump from Thermo Scientific (San Jose, CA, United States) equipped with an autosampler. The injection volume was 10 μL. Separation of lipid classes was achieved under HILIC conditions using an Accucore HILIC column (150 mm × 2.1 mm i. d., 2.6 µm, Thermo). The column was maintained at 40°C. The mobile phases consisted of (A) ACN/H_2_O (99:1, v/v) containing 10 mM ammonium acetate and (B) ACN/H_2_O (50:50, v/v) containing 10 mM ammonium acetate. The solvent-gradient system of the analytical pump was as follows: 0 min 100% A, 10 min 92% A, 40 min 50% A, 41–60 min 100% A. The flow rate was set to 500 μL.min−1. In order to guarantee analytical performance, Quality QC were used every eight test sample. HR-MS analyses of phospholipids were carried out using the Orbitrap FusionTM (Thermo Scientific, United States) Mass Spectrometer equipped with an EASY-MAX NGTM Ion Source (H-ESI). H-ESI source parameters were optimized and set as follows: Ion transfer tube temperature of 285°C, sheath gas flow rate of 35 au, auxiliary gas flow rate of 25 au, sweep gas of one au, and vaporizer temperature of 370°C. Positive and negative ions were monitored alternatively by switching polarity approach with a spray voltage set to 3500 V in positive and negative ion modes. The Orbitrap mass analyzer was employed to obtain all mass spectra in full scan mode with a mass range of 200–2000 Da, and a target resolution of 120,000 (FWHM at m/z 200). All MS data were recorded using a max injection time of 50 ms, automated gain control (AGC) at 4.105 and one microscan. An Intensity Threshold filter of 1.103 counts was applied. For MS/MS analyses, High-energy Collisional Dissociation (HCD) was employed for the fragmentation of PL species with optimized stepped collision energy of 30% (±5%). The linear ion trap (LIT) was used to acquire spectra for fragment ions in data-dependent mode. The AGC target was set to 2.104 with a max injection time of 50 ms. All MS and MS/MS data were acquired in the profile mode. The Orbitrap Fusion was controlled by XcaliburTM 4.1 software. The identification of PL species was performed, using the data of high accuracy and the information collected from fragmentation spectra (tolerance 5 ppm for MS1 and 20 ppm for MS2), with the help of the LipidSearchTM software and the LIPID MAPS^®^ database (https://www.lipidmaps.org/).

Relative quantification of the abundances of choline glycerophospholipids (ChoGpl) [including phosphatidyl cholines (PtdCho) and plasmenyl cholines (choline plasmalo gens or PlsCho)] and ethanolamine glycerophospholipids (EtnGpl) [including phosphatidylethanolamines (PtdEtn) and plasmenylethanolamines (ethanolamine plasmalogens or PlsEtn)] molecular species between samples was performed in the high resolution MS1 mode (positive for ChoGpl, negative for EtnGpl) by normalization of targeted phospholipid ion peak areas to the PtdCho14:0/14:0 or PtdEtn14:0/14:0 internal standards, respectively. Due to the lack of available lipid standards representing individual molecular species of EtnGpl and ChoGpl, the abundances of ChoGpl and EtnGpl molecular species was reported as the percentage of the total ChoGpl or EtnGpl ion abundance, respectively (after normalization on the PtdCho14:0/14:0 and PtdEtn14:0/14:0 internal standards).

### Statistical analyses

Statistical analysis was performed using GraphPad Prism v6.05 (GraphPad Software, San Diego, CA, United States), XLSTAT v2018.02.50494 (Addinsoft, Paris, France), and R Project v4.0.2 (Revolutions). Quantitative data were expressed as median and interquartile range [IQR]. The groups were compared using the nonparametric Mann-Whitney test for quantitative variables and the Chi-2 test or Fisher exact test for qualitative variables. The Benjamini-Hochberg false discovery adjusted *p*-value was applied to correct for multiple testing. Linear regression analyses were used to determine the R-squared (R^2^) correlation coefficient values. Spearman correlations were carried out to compare the levels of individual or total levels of phospholipids and ratios as a function of GA. A *p*-value lower than 0.05 was considered as statistically significant and the tests were two-tailed.

## Results

### Characteristics of the population

The characteristics of the population are presented in Table 1. As described before (Pallot et al., 2019), fifty-eight preterm infants born before 29 weeks of GA were included in the study. Six infants died and the mortality rate was 11.5%. The final population included five sets of heterozygote twins. Blood samples were obtained from 52 infants at a median time of 12 h and a maximum time of 48 h after birth. No difference was observed between the ROP and the no-ROP groups for sampling time, gender, use of erythropoietin (EPO) and cerebral hemorrhage. ROP was associated with significantly higher sepsis and red blood cell transfusion (*p* = 0.023 and *p* = 0.025, respectively), lower GA and birth weight (*p* < 0.001 and *p* = 0.001, respectively) and higher duration of mechanical ventilation (*p* < 0.001). The mean follow-up for ROP screening was 11.3 ± 4.5 weeks of life.

**TABLE 1:** Main characteristics of the OmegaROP population.

	ROP *n* = 27	No-ROP *n* = 25	*p*-value
Sampling time (h)	12 [12–21]	12 [12–30]	0.056
Male	14 (51.8)	12 (48.0)	0.781
Gestational age (weeks)	26.5 [25.5–27.1]	27.6 [27.1–28.4]	<0.001
Birth weight (g)	815 [735–967]	1020 [870–1160]	0.001
ROP	27 (100)	–	–
ROP treated	3 (11.1)	–	–
ROP detection (weeks)	8.2 [6.6–9.5]	–	–
Mechanical ventilation (days)	11.0 [5.5–16.5]	2.0 [1.0–7.0]	<0.001
Sepsis	16 (59.2)	7 (28.0)	0.023
Erythropoietin use	18 (66.6)	15 (60.0)	0.617
RBC transfusion	17 (62.9)	8 (32.0)	0.025
Cerebral haemorrhage	13 (48.1)	12 (48.0)	0.991

Continuous variables are expressed as median [IQR], categorical variables are expressed as No (%).

ROP: Retinopathy of prematurity; RBC: Red blood cells.

*p*-values in bold indicate a statistically significant difference (*p* < 0.05).

The mean number of screening examinations was 3.6 ± 2.0 per infant. The incidence of ROP was 51.9%, including three cases of type 1 ROP (11.1%) and 24 cases of type 2 ROP (88.9%). Subjects with type 1 ROP underwent laser therapy on both eyes. No intravitreal injection of bevacizumab was performed. Twenty-six ROP cases were observed in zone 2 (96.3%) and one ROP case was observed in zone 3 (3.7%). We did not observe any ROP in zone 1. One case of ROP was stage 1 (3.7%), 19 cases were stage 2 (70.4%) and seven cases were stage 3 (25.9%). Four subjects were classified as a “plus” stage and three of them underwent laser treatment.

### Individual phospholipid species distribution in erythrocytes

**FIGURE 1:**
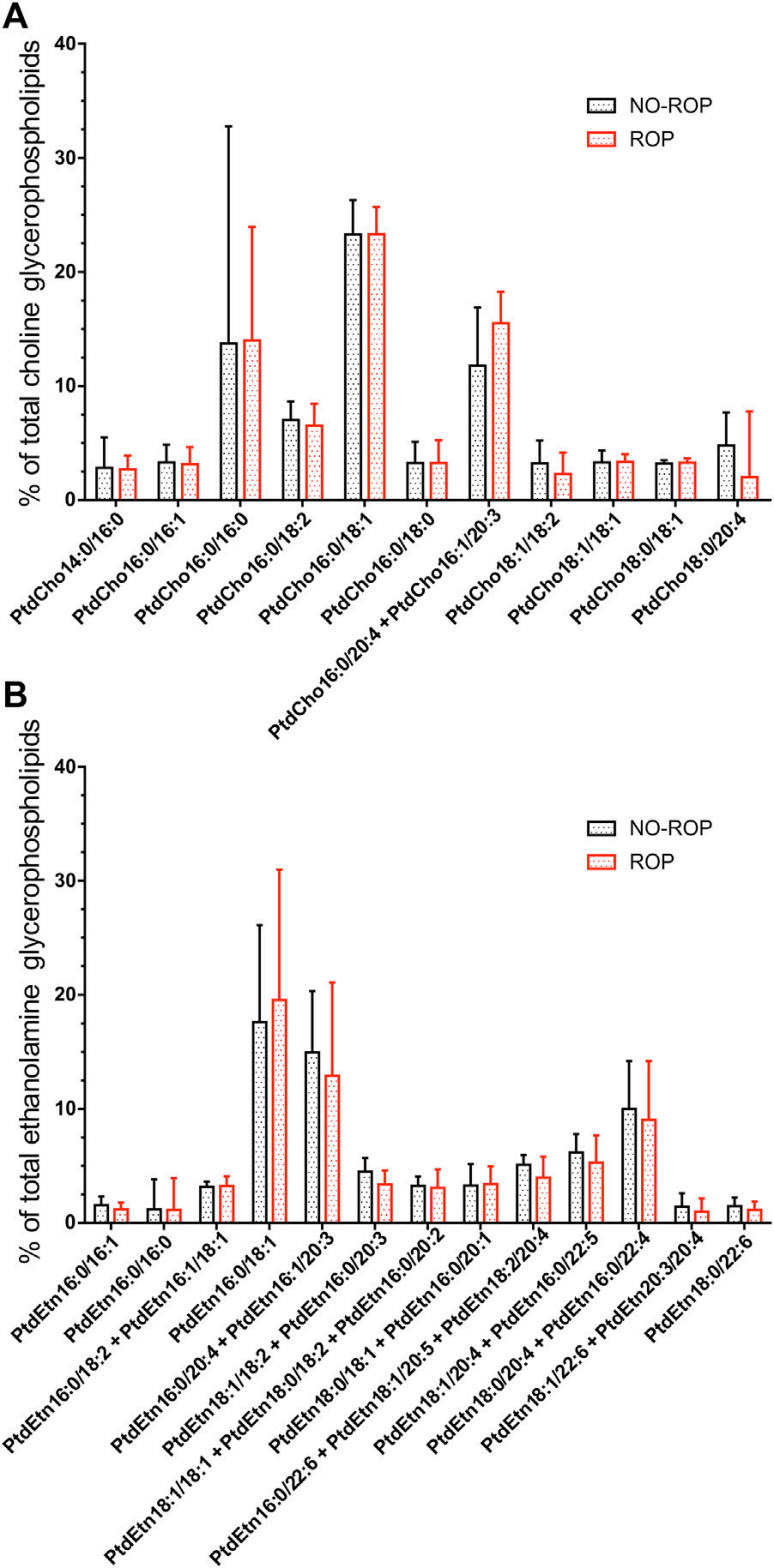
Concentrations of major individual ChoGpl species **(A)** and major EtnGpl species **(B)** of red blood cells of preterm infants without or with retinopathy of prematurity (% of total ChoGpl or % of total EtnGpl). No significant difference was observed between no-ROP and ROP groups for any specie. Abbreviations of individual phospholipid species are as follows: Position on the glycerol backbone as shown as sn-1/sn-2 of the fatty alcohol radicals (abbreviated as number of carbons: Number of double bonds). PtdCho: Phosphatidylcholine; PtdEtn: Phosphatidylethanolamine; ROP: Retinopathy of prematurity.

No significant difference was observed between groups in the proportions of individual EtnGpl and ChoGpl species as well as in total levels of PtdCho, PlsCho, PtdEtn, and PlsEtn (Figure 1 and Supplementary Table S1). The predominant species were PtdCho16:0/18:1, PtdCho16:0/16:0 and PtdCho16:0/20:4 + PtdCho16:1/20:3 for ChoGpl and PtdEtn16:0/18:1, PtdEtn16:0/20:4 + PtdEtn16:1/20:3, and PtdEtn18:0/20:4 + 16:0/22:4 for EtnGpl. The most represented plasmalogen species were PlsCho16:0/16:1, PlsCho18:0/16:0 and PlsCho18:1/18:2 for the choline subgroup and PlsEtn18:1/22:4 and PlsEtn18:0/22:4 the ethanolamine subgroup. The wide interquartile ranges confirmed the high interindividual variability previously observed for the fatty acid concentrations (Pallot et al., 2019).

### Associations between gestational age and erythrocyte phospholipid species

**FIGURE 2:**
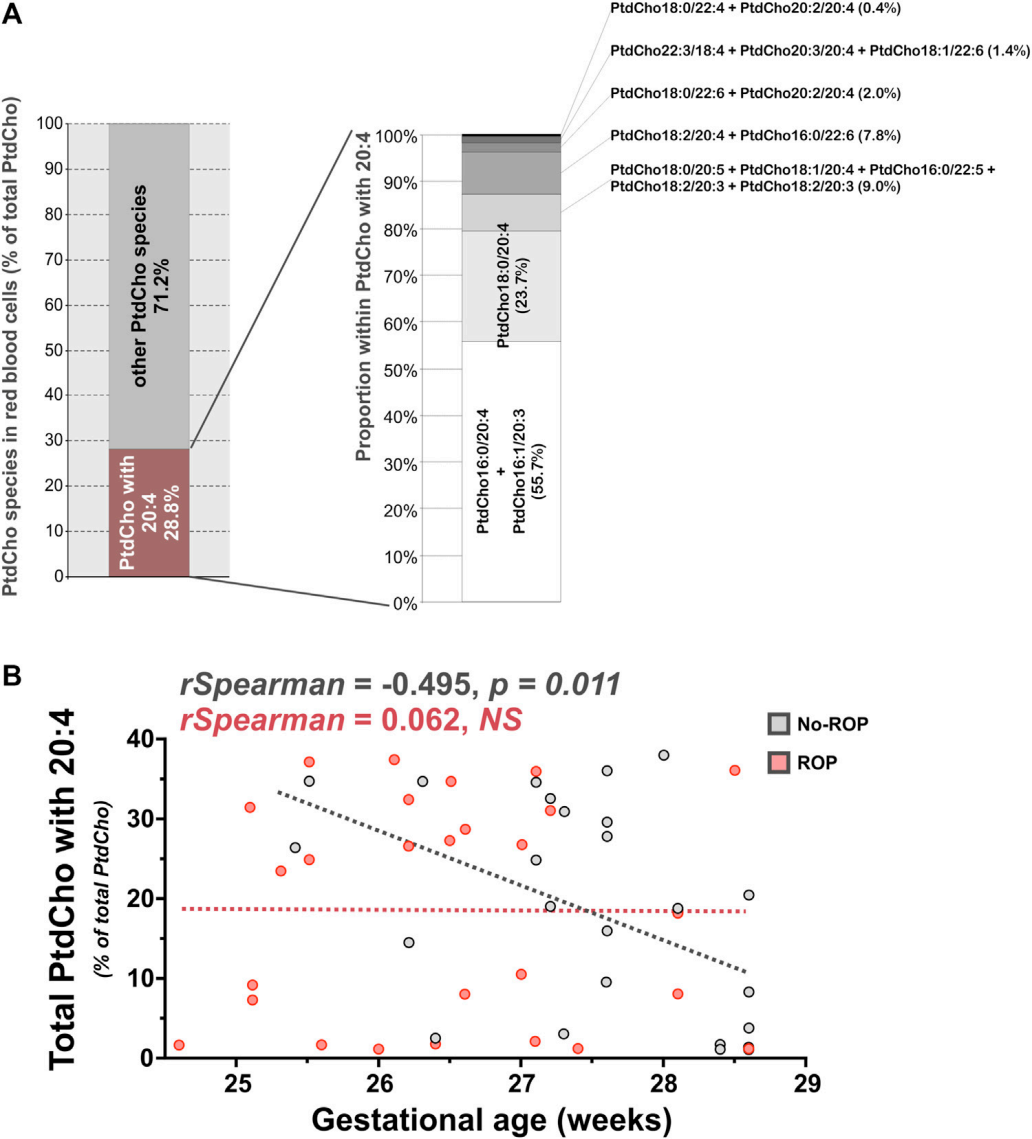
PtdCho with ARA are associated with GA in preterm infants without ROP. **(A)** PtdCho with ARA (20:4) accounted for 28.8% of total retinal PtdCho species. **(B)** The sum of PtdCho species carrying ARA was negatively associated to GA in erythrocytes of preterm infants of the no-ROP group (rho = −0.495, *p* = 0.011). Abbreviations of individual PtdCho species are as follows: Position on the glycerol backbone as shown as sn-1/sn-2 of the fatty alcohol radicals (abbreviated as number of carbons: Number of double bonds). PtdCho: Phosphatidylcholine; ROP: Retinopathy of prematurity.

As in our previous work on the OmegaROP cohort and considering the results of the principal component analysis showing an interaction between GA and lipid data (Pallot et al., 2019), we checked for Spearman correlations between GA and individual phospholipid species (Table 2). In order to identify the phospholipid origin of the differential accumulation of ARA and/or DHA in subjects with or without ROP, we focused our attention on phospholipids carrying n-6 and n-3 PUFAs. Even if the correlations are weak, total ChoGpl and EtnGpl carrying n-6 PUFAs were negatively associated with GA in the no-ROP group (rho = −0.485, *p* = 0.013 and rho = −0.477, *p* = 0.015 for PtdCho + PlsCho and PtdEtn + PlsEtn, respectively). Within the choline subgroup, this negative association was weak but significant only for total PtdCho species esterified with n-6 PUFAs (rho = −0.509, *p* = 0.009), but not for PlsCho. PtdCho species carrying ARA are likely to contribute to this finding since total PtdCho species carrying ARA were also negatively associated with GA in the no-ROP group (rho = −0.495, *p* = 0.011, Panel B Figure 2), and particularly the individual PtdCho16:0/20:4, PtdCho18:1/20:4, and PtdCho18:0/20:4 species (Table 3). PC carrying ARA represented 28.8% of total PtdCho species (Panel A on Figure 2). Within EtnGpl, only PlsEtn carrying n-6 PUFAs were negatively associated with GA (rho = −0.587, *p* = 0.002). Although the correlation is not strong, PlsEtn18:1/20:4 may be a significant contributor to this observation (rho = −0.395, *p* = 0.049). No significant association with GA was observed for PtdEtn species.

**TABLE 2:** Spearman correlations between gestational age and erythrocyte ChoGpl and EtnGpl esterified with n-6 and/or n-3 PUFAs in preterm infants with or without retinopathy of prematurity.

	No-ROP *n* = 25					ROP *n* = 27				
	Median	[IQR]^a^	rho	R^2^	*p*-value	Median	[IQR]	rho	R^2^	*p*-value
Total PtdCho and PlsCho with n-6 PUFAs	31.21	[9.27–43.84]	−0.485	0.202	0.013	36.86	[7.09–45.82]	0.079	0.001	0.693
Total PtdCho and PlsCho with n-3 PUFAs	1.81	[0.55–4.46]	−0.375	0.109	0.064	2.03	[0.58–4.50]	−0.160	0.010	0.422
Total PtdEtn and PlsEtn with n-6 PUFAs	64.70	[28.52–70.89]	−0.477	0.200	0.015	67.43	[25.10–69.83]	0.050	0.016	0.801
Total PtdEtn and PlsEtn with n-3 PUFAs	21.64	[15.28–24.81]	−0.117	0.004	0.575	19.62	[13.88–24.64]	−0.380	0.084	0.034
Total PtdCho with n-6 PUFAs	31.10	[9.27–42.67]	−0.509	0.208	0.009	36.56	[6.72–43.73]	0.122	0.002	0.544
Total PlsCho with n-6 PUFAs	0.80	[0.19–2.18]	−0.191	0.028	0.359	0.60	[0.05–2.07]	−0.198	0.029	0.319
Total PtdCho with n-3 PUFAs	1.55	[<0.01–4.04]	−0.391	0.126	0.052	2.03	[<0.01–3.89]	−0.011	0.000	0.953
Total PlsCho with n-3 PUFAs	0.16	[<0.01–0.42]	−0.093	0.000	0.657	0.28	[<0.01–0.83]	−0.352	0.105	0.071
Total PtdEtn with n-6 PUFAs	53.05	[28.52–63.07]	−0.311	0.123	0.129	45.36	[25.04–65.50]	0.211	0.050	0.288
Total PlsEtn with n-6 PUFAs	0.77	[0.33–12.68]	−0.587	0.075	0.002	0.58	[0.13–18.47]	0.027	0.019	0.892
Total PtdEtn with n-3 PUFAs	18.25	[13.88–21.64]	0.025	0.000	0.904	17.32	[12.68–19.94]	−0.260	0.063	0.189
Total PlsEtn with n-3 PUFAs	<0.01	[<0.01–3.26]	−0.326	0.062	0.111	<0.01	[<0.01–5.08]	−0.215	0.035	0.281
Total PtdCho with ARA	19.00	[3.06–30.88]	−0.495	0.204	0.011	26.57	[1.74–31.86]	0.062	0.000	0.756
Total PlsCho with ARA	<0.01	[<0.01–1.01]	−0.340	0.068	0.096	<0.01	[<0.01–1.04]	−0.104	0.003	0.603
Total PtdEtn with ARA	38.24	[17.95–46.04]	−0.319	0.130	0.119	32.92	[14.28–47.04]	0.243	0.039	0.221
Total PlsEtn with ARA	<0.01	[<0.01–9.33]	−0.286	0.066	0.164	<0.01	[<0.01–14.25]	−0.110	0.020	0.583
Total PtdCho with DHA	1.55	[<0.01–3.14]	−0.359	0.128	0.077	2.03	[<0.01–2.71]	−0.016	0.000	0.936
Total PlsCho with DHA	<0.01	[<0.01–0.16]	−0.308	0.053	0.133	<0.01	[<0.01–0.17]	−0.131	0.010	0.514
Total PtdEtn with DHA	8.93	[7.43–11.97]	0.189	0.069	0.364	8.77	[5.97–10.48]	−0.132	0.097	0.509
Total PlsEtn with DHA	<0.01	[<0.01–1.32]	−0.338	0.067	0.098	<0.01	[<0.01–2.04]	−0.206	0.028	0.300
Total PtdCho with n-6 PUFAs/total PtdCho with n-3 PUFAs	10.57	[<0.01–16.63]	−0.286	0.039	0.165	10.96	[<0.01–16.79]	0.312	0.090	0.112
Total PtdCho with ARA/total PtdCho with DHA	10.27	[<0.01–11.06]	−0.410	0.127	0.041	11.13	[<0.01–12.46]	0.242	0.052	0.222
Total PlsCho with n-6 PUFAs/total PlsCho with n-3 PUFAs	<0.01	[<0.01–2.27]	−0.355	0.062	0.081	<0.01	[<0.01–1.44]	−0.063	0.001	0.752
Total PlsCho with ARA/total PlsCho with DHA	<0.01	[<0.01–6.33]	−0.390	0.106	0.053	<0.01	[<0.01–6.27]	−0.088	0.001	0.661
Total PtdEtn with n-6 PUFAs/total PtdEtn with n-3 PUFAs	2.99	[2.57–4.11]	−0.072	0.027	0.731	3.19	[2.62–4.31]	0.420	0.032	0.029
Total PtdEtn with ARA/total PtdEtn with DHA	4.33	[3.88–5.15]	−0.239	0.033	0.249	4.58	[3.84–4.91]	0.366	0.057	0.060
Total PlsEtn with n-6 PUFAs/total PlsEtn with n-3 PUFAs	<0.01	[<0.01–2.71]	−0.308	0.059	0.133	<0.01	[<0.01–2.83]	0.886	0.269	0.006
Total PlsEtn with ARA/total PlsEtn with DHA	<0.01	[<0.01–5.70]	−0.296	0.056	0.150	<0.01	[<0.01–5.79]	0.843	0.214	0.011

a IQR: Interquartile range; median and IQR, values are given as % of total choline phospholipids or % of total ethanolamine phospholipids. ARA: Arachidonic acid; DHA, docosahexaenoic acid; PtdCho: Phosphatidylcholine; PlsCho: Plasmenylcholine; PtdEtn: Phosphatidylethanolamine; PlsEtn: Plasmenylethanolamine; ROP: Retinopathy of prematurity.

**TABLE 3:** Spearman correlations between gestational age and erythrocyte individual phospholipids species esterified with n-6 and/or n-3 PUFAs in preterm infants with or without retinopathy of prematurity.

	[M + H]+ or [M-H]-^a^	No-ROP *n* = 25			ROP *n* = 27		
		rho	R^2^	*p*-value	rho	R^2^	*p*-value
Phosphatidylcholine species							
PtdCho16:0/20:4 + PtdCho16:1/20:3	782.5695	−0.511	0.225	0.009	0.013	0.001	0.945
PtdCho18:2/20:4 + PtdCho16:0/22:6	806.5695	−0.346	0.127	0.089	−0.016	0.000	0.936
PtdCho18:0/20:5 + PtdCho18:1/20:4 + PtdCho16:0/22:5 + PtdCho18:2/20:3 + PtdCho20:3/18:2	808.5851	−0.479	0.165	0.015	−0.023	0.033	0.906
PtdCho18:0/20:4	810.6008	−0.517	0.199	0.008	0.183	0.003	0.360
PtdCho20:5/22:6	828.5538	−0.338	0.073	0.097	−0.123	0.008	0.538
PtdCho22:3/18:4 + PtdCho20:3/20:4 + PtdCho18:1/22:6	832.5851	−0.304	0.069	0.138	−0.58	0.003	0.772
PtdCho18:0/22:6 + PtdCho20:2/20:4	834.6008	−0.331	0.068	0.105	−0.088	0.005	0.660
PtdCho18:0/22:4 + PtdCho20:0/20:4	838.6321	−0.350	0.087	0.086	−0.045	0.002	0.822
Plasmenylcholine species							
PlsCho18:1/20:4 + PlsCho16:1/22:4	792.5902	−0.352	0.057	0.083	−0.122	0.012	0.542
PlsCho18:0/20:4 + PlsCho16:0/22:4	794.6058	−0.336	0.070	0.100	−0.104	0.001	0.603
PlsCho18:0/22:6	818.6058	−0.308	0.053	0.133	−0.131	0.010	0.514
Phosphatidylethanolamine species							
PtdEtn16:0/20:4 + PtdEtn16:1/20:3	738.5079	−0.298	0.111	0.147	0.196	0.071	0.327
PtdEtn16:0/22:6 + PtdEtn18:1/20:5 + PtdEtn18:2/20:4	762.5079	−0.213	0.115	0.306	0.188	0.035	0.347
PtdEtn18:1/20:4 + PtdEtn16:0/22:5	764.5236	−0.432	0.215	0.030	0.082	0.010	0.683
PtdEtn18:0/20:4 + PtdEtn16:0/22:4	766.5392	−0.344	0.130	0.091	0.200	0.065	0.315
PtdEtn20:4/20:4 + PtdEtn18:2/22:6	786.5079	0.001	0.056	0.992	−0.185	0.128	0.354
PtdEtn18:1/22:6 + PtdEtn20:3/20:4	788.5236	−0.259	0.051	0.210	0.106	0.004	0.596
PtdEtn18:0/22:6	790.5392	−0.306	0.106	0.136	0.083	0.005	0.678
PtdEtn18:0/22:4 + PtdEtn20:0/20:4	794.5705	−0.282	0.148	0.171	0.314	0.062	0.110
PtdEtn20:4/22:6	810.5079	−0.047	0.002	0.821	−0.285	0.103	0.148
PtdEtn22:6/22:6	834.5079	0.439	0.168	0.027	−0.011	0.113	0.954
Plasmenylethanolamine species							
PlsEtn16:0/20:4	722.5130	−0.376	0.069	0.063	−0.179	0.018	0.369
PlsEtn16:0/22:6	746.5130	−0.343	0.070	0.092	−0.193	0.022	0.333
PlsEtn18:1/20:4	748.5287	−0.395	0.065	0.049	−0.214	0.019	0.283
PlsEtn18:0/20:4 + PlsEtn16:0/22:4	750.5443	−0.298	0.066	0.146	−0.117	0.021	0.560
PlsEtn18:1/22:6	772.5287	−0.326	0.064	0.111	−0.223	0.033	0.262
PlsEtn18:0/22:6 + PlsEtn18:1/22:5	774.5443	−0.326	0.059	0.111	−0.216	0.040	0.277

^a^[M + H]+ for PtdCho and PlsCho, and [M + H]+ for PtdEtn and PlsEtn species. PtdCho: Phosphatidylcholine; PlsCho: Plasmenylcholine; PtdEtn: Phosphatidylethanolamine; PlsEtn: Plasmenylethanolamine; ROP: Retinopathy of prematurity. abrAbbreviationsof individual PtdCho, PlsCho, PtdEtn, and PlsEtn species are as follows: Position on the glycerol backbone as shown as sn-1/sn-2 of the fatty acid and fatty alcohol radicals (abbreviated as number of carbons: Number of double bonds).

In the no-ROP group, no significant association between GA and phospholipids carrying n-6 PUFAs was observed (Tables 2 and 3). Only a weak significant negative association was observed between GA and total EtnGpl esterified with n-3 PUFAs (rho = −0.380, *p* = 0.034). Probably as a consequence, the ratios of total PtdEtn with n-6 PUFAs to total PtdEtn with n-3 PUFAs, total PlsEtn with n-6 PUFAs to total PlsEtn with n-3 PUFAs, and total PtdEtn with ARA to total PtdEtn with DHA were impacted and positively associated with GA in the ROP group (rho = 0.420, 0.886, and 0.843, *p* = 0.029, 0.006, and 0.011, respectively; Table 2).

## Discussion

This study characterizes the phospholipid composition of erythrocytes in preterm infants born before 29 weeks GA. The clinical characteristics of our population were comparable to those of several studies. Indeed, the incidence of ROP was high (51.9%) and in agreement with other very-low-GA populations (Austeng, Kallen, Ewald, Jakobsson, & Holmstrom, 2009).

Our data show a weak but negative association between ChoGpl and EtnGpl carrying n-6 PUFAs with GA in the no-ROP group, while no significant association was observed in the ROP group. These findings are in the line with our previous reports related to the OmegaROP study (Pallot et al., 2019). Considering that ChoGpl and EtnGpl species represent more than 90% of total phospholipids in erythrocytes (Acar et al., 2007; Acar et al., 2009; Acar et al., 2012), we assumed that the changes observed in their concentrations would be a reliable indicator of the whole phospholipid pool in erythrocytes. However, further analyses on phosphatidylserines and phosphatidylinositols could be of interest to draw a more complete picture of erythrocyte phospholipidome alterations in ROP.

In preterm infants who will not develop ROP, erythrocyte relative levels of phospholipids carrying ARA decrease as GA increases, while no change was observed in preterm infants who will develop ROP. This negative correlation mostly relies on PtdCho species and more specifically on individual PtdCho16:0/20:4, PtdCho18:1/20:4, and PtdCho18:0/20:4 species. This finding suggests an *in utero* accumulation of ARA in erythrocytes of preterm infants that will develop ROP relatively to those that will not develop ROP. This GA-related change is in line with the finding of Bernhard and collaborators that revealed a remodeling in plasma PtdCho species esterified with ARA in early preterm infants (Bernhard et al., 2016). They showed that the plasma PtdCho ARA to DHA ratio in preterm infants of less than 33 weeks GA largely exceeds that of term infants, thus likely contributing to the prematurity-related impaired overall health. The data we have obtained in 25- to 28-weeks GA newborns is then in agreement with this observation. However, it is important to keep in mind that the biological materials used in these two studies were different, making that further analyses on plasma PtdCho of our subjects would be of interest.

Whether the relative accumulation of ARA in erythrocytes is associated to the onset of ROP remains to be further investigated. Indeed, on one hand it is very clear that together with DHA, ARA is essential during gestation and early postnatal life for an optimal development of the infant (Tai, Wang, & Chen, 2013; Koletzko et al., 2020; Bernhard, Poets, & Franz, 2021). On the other hand, ARA is known to play a pivotal role in the promotion of inflammation, especially through its eicosanoid derivatives such as prostaglandin E2 (PGE2). PGE2 is known to be involved in abnormal angiogenesis and in the pathogenesis of proliferative retinopathies such as ROP (Yanni, Barnett, Clark, & Penn, 2009; Hartnett & Penn, 2012; Schoenberger et al., 2012; Xie et al., 2021). Accordingly, other studies have shown that inflammation is a significant risk factor for developing ROP (Holm et al., 2017; Rivera et al., 2017). Finally, it is recognized that ARA competes with DHA, the latter being known to inhibit retinal neovascularization in a mouse model of ROP (Connor et al., 2007). DHA and ARA are the most prevalent PUFAs in the human retina (Bretillon et al., 2008; Acar et al., 2012). As a subtle equilibrium in their needs may exist within this tissue, we cannot exclude that minor variations in the ARA to DHA ratio may impact retinal physiology.

In accordance with our previous observation on the same cohort (Pallot et al., 2019), our data display a high interindividual variability. This is especially true for ARA whose levels in total lipids were of 13.48 ± 7.29% and 13.86 ± 7.63% of total fatty acids in the no-ROP and ROP groups, respectively (Pallot et al., 2019). The present study shows that ARA levels were low especially in PtdCho, and independently from the development of ROP. Although extensive analyses, we were unable to connect these very low ARA levels to any other variable, including GA, blood sampling time after birth or any other parameter related to the infants’ health. Since we have used analytical QCs and as we have repeated our analyses several times, we believe that these changes are of physiologic origin and not the result of analytical bias. The probable physiological origin of these changes is reinforced by similar observations showing very-low levels of ARA (Glen et al., 1994; Khan et al., 2002) or even ARA levels close to zero (Li et al., 2022) in erythrocytes of human subjects with neurologic disorders or of infants with severe malnutrition (Smit et al., 1997). Such data may suggest a marked dysregulation of erythrocyte membrane fatty acids in some infants. It can be the result of either increased phospholipase A2 activity and/or enhanced lipid peroxidation. Free radical oxidation during technical steps of sample handling and lipid analysis can also be at the origin of the degradation of PUFAs such as AA, but also that of plasmalogens. To avoid such biases in our study, we took care to take a number of precautions starting from immediate processing of the blood sample after venipuncture, to the isolation and washing of red cells at a temperature of 4°C, their immediate storage at −80°C, and the use of the Moilanen & Nikkari procedure for lipid extraction. This last methodology doesn’t use acidic conditions that are known to be deleterious for plasmalogens. Another hypothesis to explain the low levels of ARA would rely on a reduced bioavailability of ARA in maternal blood and/or its abnormal placental transfer. Further investigation is needed to better understand the origin of these modifications.

Circulating lipids are also subject to a postnatal remodeling. Indeed, several studies documented an increase in the plasma and erythrocyte levels of linoleic acid (the dietary precursor of ARA) and a decrease in the levels of ARA during the first weeks of life as a consequence of a LA-rich nutrition (Bernhard et al., 2014; Bockmann et al., 2021). Considering that the birth to blood sampling delay in our study exceeded 24 h in some cases, we cannot exclude the influence of the source of lipid (parenteral versus enteral nutrition) on the lipid data, especially in infants with lower GA for which the parenteral lipid supply is higher. Nevertheless, we did not find any association between erythrocyte lipids (and especially PtdCho16:0/20:4) and the sampling time in the subjects of our cohort (data not shown).

Interestingly, the n-6 to n-3 PUFA ratio in erythrocytes seems to display a similar pattern in the no-ROP group when considering PlsEtn species, as PlsEtn carrying n-6 PUFAs were negatively associated with GA (rho = −0.587, *p* = 0.002), with PlsEtn18:1/20:4 being the most significant contributor to this observation (rho = −0.395, *p* = 0.049). Considering that erythrocyte lipid composition could represent a reliable indicator of the lipid composition of the retina in newborns (Carlson, Carver, & House, 1986; Makrides, Neumann, Byard, Simmer, & Gibson, 1994; Sarkadi-Nagy et al., 2004), we may speculate that retinal lipids display similar modifications in ARA, DHA and plasmalogen levels of newborns developing ROP. On the contrary, infants that will develop ROP seem to accumulate n-6 PUFAs in their plasmalogens as the ratio of PlsEtn carrying n-6 PUFAs to PlsEtn carrying n-3 PUFAs is strongly positively associated to GA (rho = 0.886, *p* = 0.006), ARA and DHA being the major contributors to this observation (rho = 0.843, *p* = 0.011). These data are in line with the ROP-like phenotype observed in plasmalogen-deficient mice (Saab et al., 2014) and then reinforce the idea that plasmalogens may participate to the pathophysiology of this disease. It would be however interesting to check whether these mice display changes in the levels of PUFAs such as ARA and/or DHA in their erythrocytes in order to strengthen the conclusions of the present paper. While PUFAs status of the newborn correlates with maternal status (Bernhard et al., 2016), and as it can be influenced by dietary supplementation after birth (Schulzke, Patole, & Simmer, 2011), plasmalogen content of tissues only relies on fetal *de novo* synthesis as no materno-fetal transfer of ether-lipids has been demonstrated so far (Das, Holmes, Wilson, & Hajra, 1992). In our study, blood samples were collected within the first 48 h of life. Considering that the erythrocyte life-span ranges from 35 to 50 days in preterm infants (Pearson, 1967), the modifications observed in the present study might be assigned to differences *in situ* lipid metabolisms even if, again, a nutritional influence cannot be excluded for PUFAs.

Several limitations must be acknowledged in our study. First, the correlation coefficients found in this study are weak and deserve more investigations. Second, our population included a limited number of patients and subjects. Third, our population only included three preterm infants with severe ROP, while this population was specifically concerned by treatments targeting vascular events. Furthermore, while ChoGpl and EtnGpl species are the main contributors to erythrocytes phospholipidome, further lipidomic analyses including phosphatidylserine and phosphatidylinositol individual phospholipid species could provide useful complementary information.

Taken together, our results seem to confirm the alterations of erythrocyte PUFA profile in preterm infants developing ROP and suggest that plasmalogens may contribute to them. Considering the importance of plasmalogens in the cellular bioavailability of PUFAs and their involvement in the vascular development of the retina, our study suggests that investigating the relationships between plasmalogen metabolism and ROP could be of particular interest to decipher the pathophysiological mechanisms driving ROP.

## Data Availability

The datasets presented in this study can be found in online repositories. The names of the repository/repositories and accession number(s) can be found below: https://www.ebi.ac.uk/metabolights/MTBLS4219/descriptors
