# Supplementary material for: Modification of erythrocyte membrane phospholipid composition in preterm newborns with retinopathy of prematurity: The omegaROP study
Source: Front Cell Dev Biol. 2022 Sep 9;10:921691. doi: 10.3389/fcell.2022.921691 (PMC9504055; doi:10.3389/fcell.2022.921691)
Supplement: Supplementary file 1 [file Table1.docx]

| **Supplementary Table 1:** Concentrations of individual phospholipid species of red blood cells of preterm infants without or with retinopathy of prematurity (% of total choline phospholipids or % of total ethanolamine phospholipids) | | | | | | | | |
| --- | --- | --- | --- | --- | --- | --- | --- | --- |
|  |  |  |  | **No-ROP** | | **ROP** | |  |
|  | **measured mass*^a^*** | **theorical mass** | **delta (ppm)** | **median** | *[IQR]^b^* | **median** | *[IQR]* | adjusted *p*-value |
| PtdCho14:0/16:0 | 706.53804 | 706.53813 | -0.13 | **2.84** | *[1.59–5.35]* | **2.69** | *[1.81-3.69]* | *0.965* |
| PtdCho15:0/16:0 | 720.55396 | 720.55378 | 0.25 | **0.80** | *[0.40-1.43]* | **0.82** | *[0.46-1.26]* | *0.965* |
| PlsCho16:0/16:1 | 720.59006 | 720.59017 | -0.14 | **1.04** | *[0.67-2.64]* | **1.05** | *[0.56-1.98]* | *0.965* |
| PtdCho16:1/16:1 | 730.53841 | 730.53813 | 0.38 | **0.10** | *[<0.01-0.15]* | **0.09** | *[<0.01-0.14]* | *0.965* |
| PtdCho16:0/16:1 | 732.55396 | 732.55378 | 0.24 | **3.31** | *[41.96-4.59]* | **3.15** | *[1.84-4.39]* | *0.965* |
| PtdCho16:0/16:0 | 734.56933 | 734.56943 | -0.13 | **13.73** | *[7.28-31.24]* | **14.00** | *[8.15-23.80]* | *0.965* |
| PlsCho16:0/18:1 | 744.59030 | 744.59017 | 0.19 | **0.06** | *[<0.01-0.20]* | **0.07** | *[<0.01-0.16]* | *0.965* |
| PtdCho15:0/18:0 | 746.56972 | 746.56943 | 0.39 | **0.50** | *[0.42-0.57]* | **0.46** | *[0.40-0.57]* | *0.965* |
| PlsCho18:0/16:0 | 746.60578 | 746.60582 | -0.04 | **0.94** | *[0.76-1.14]* | **0.86** | *[0.59-1.19]* | *0.965* |
| PtdCho16:0/18:2 | 758.56993 | 758.56943 | 0.66 | **7.03** | *[4.07-8.64]* | **6.52** | *[4.02-8.29]* | *0.965* |
| PtdCho16:0/18:1 | 760.58529 | 760.58508 | 0.28 | **23.32** | *[18.27-25.66]* | **23.33** | *[17.99-25.58]* | *0.965* |
| PtdCho16:0/18:0 | 762.60088 | 762.60078 | 0.13 | **3.28** | *[2.62-5.09]* | **3.27** | *[2.53-5.14]* | *0.965* |
| PlsCho18:1/18:3 | 766.57441 | 766.57452 | -0.14 | **<0.01** | *[<0.01-0.33]* | **0.24** | *[<0.01-0.64]* | *0.965* |
| PlsCho18:1/18:2 | 768.59024 | 768.59017 | 0.10 | **0.89** | *[<0.01-1.14]* | **0.73** | *[<0.01-1.01]* | *0.965* |
| PlsCho18:1/18:0 | 772.62159 | 772.62147 | 0.16 | **0.21** | *[<0.01-0.33]* | **0.16** | *[<0.01-0.33]* | *0.965* |
| unknown | 774.60116 | 774.60073 | 0.56 | **0.30** | *[<0.01-0.38]* | **0.33** | *[<0.01-0.48]* | *0.965* |
| PlsCho18:0/18:0 | 774.63713 | 774.63712 | 0.02 | **0.10** | *[<0.01-0.23]* | **0.11** | *[<0.01-0.19]* | *0.965* |
| PtdCho16:0/20:4 + PtdCho16:1/20:3 | 782.57001 | 782.56943 | 0.74 | **11.80** | *[1.92-16.75]* | **15.50** | *[1.10-17.56]* | *0.965* |
| PtdCho18:1/18:2 | 784.58409 | 784.58508 | -1.26 | **3.24** | *[1.96-5.21]* | **2.30** | *[1.35-4.12]* | *0.965* |
| PtdCho18:1/18:1 | 786.60035 | 786.60073 | -0.48 | **3.31** | *[1.53-4.34]* | **3.34** | *[2.43-3.98]* | *0.965* |
| PtdCho18:0/18:1 | 788.61595 | 788.61638 | -0.54 | **3.22** | *[1.59-3.44]* | **3.30** | *[2.05-3.67]* | *0.965* |
| PtdCho18:0/18:0 | 790.63218 | 790.63208 | 0.12 | **<0.01** | *[<0.01-0.40]* | **0.30** | *[<0.01-0.42]* | *0.965* |
| PlsCho18:1/20:4 + PlsCho16:1/22:4 | 792.59023 | 792.59017 | 0.09 | **<0.01** | *[<0.01-0.17]* | **<0.01** | *[<0.01-0.23]* | *0.965* |
| PlsCho18:0/20:4 + PlsCho16:0/22:4 | 794.60585 | 794.60582 | 0.04 | **<0.01** | *[<0.01-0.82]* | **<0.01** | *[<0.01-0.85]* | *0.965* |
| PlsCho18:0/20:3 + PlsCho18:1/20:2 | 796.62133 | 796.62147 | -0.17 | **<0.01** | *[<0.01-0.70]* | **<0.01** | *[<0.01-0.66]* | *0.965* |
| unknown | 804.55255 | 804.55378 | -1.53 | **0.32** | *[0.19-0.52]* | **0.36** | *[0.26-0.50]* | *0.965* |
| PtdCho18:2/20:4 + PtdCho16:0/22:6 | 804.58897 | 804.59017 | -1.49 | **1.55** | *[<0.01-3.09]* | **2.03** | *[<0.01-2.66]* | *0.965* |
| PtdCho18:0/20:5 + PtdCho18:1/20:4 + PtdCho16:0/22:5 +  PtdCho18:2/20:3 + PtdCho18:2/20:3 | 808.58463 | 808.58508 | -0.55 | **1.29** | *[0.37-2.67]* | **1.26** | *[0.25-2.86]* | *0.965* |
| PtdCho18:0/20:4 | 810.60081 | 810.60073 | 0.10 | **4.80** | *[0.77-7.38]* | **2.01** | *[0.45-7.54]* | *0.965* |
| PtdCho18:0/20:2 | 814.63219 | 814.63208 | 0.13 | **0.03** | *[<0.01-0.16]* | **0.10** | *[<0.01-0.15]* | *0.965* |
| PlsCho18:0/22:6 | 818.60557 | 818.60582 | -0.30 | **<0.01** | *[<0.01-0.16]* | **<0.01** | *[<0.01-0.17]* | *0.965* |
| PlsCho18:1/22:4 + PlsCho18:0/22:5 | 820.62112 | 820.62147 | -0.42 | **<0.01** | *[<0.01-0.19]* | **<0.01** | *[<0.01-0.19]* | *0.965* |
| PlsCho18:0/22:4 | 822.63690 | 822.63712 | -0.26 | **0.19** | *[0-14-0.39]* | **0.18** | *[0.05-0.25]* | *0.965* |
| PlsCho18:1/22:2 | 824.65295 | 824.65277 | 0.22 | **<0.01** | *[<0.01-0.13]* | **<0.01** | *[<0.01-0.12]* | *0.965* |
| PlsCho18:1/22:1 | 826.66857 | 826.66842 | 0.18 | **<0.01** | *[<0.01-0.04]* | **<0.01** | *[<0.01-0.03]* | *0.965* |
| PtdCho20:5/22:6 | 828.55646 | 828.55378 | 3.24 | **<0.01** | *[<0.01-0.05]* | **<0.01** | *[<0.01-0.05]* | *0.965* |
| unknown | 830.56924 | 830.56943 | -0.23 | **0.33** | *[0.25-0.59]* | **0.36** | *[0.19-0.59]* | *0.965* |
| PtdCho22:3/18:4 + PtdCho20:3/20:4 + PtdCho18:1/22:6 | 832.58462 | 832.58508 | -0.56 | **<0.01** | *[<0.01-0.64]* | **<0.01** | *[<0.01-0.67]* | *0.965* |
| PtdCho18:0/22:6 + PtdCho20:2/20:4 | 834.60050 | 834.60073 | -0.27 | **<0.01** | *[<0.01-0.04]* | **<0.01** | *[<0.01-1.01]* | *0.965* |
| PtdCho18:0/22:4 + PtdCho20:0/20:4 | 838.63147 | 838.63203 | -0.67 | **<0.01** | *[<0.01-0.05]* | **<0.01** | *[<0.01-0.23]* | *0.965* |
| unknown | 854.56885 | 854.56943 | -0.67 | **0.05** | *[<0.01-0.12]* | **0.07** | *[<0.01-0.12]* | *0.965* |
| PtdCho20:0/22:1 | 872.71041 | 872.71028 | 0.14 | **0.01** | *[<0.01-0.04]* | **0.03** | *[<0.01-0.05]* | *0.965* |
| Total PtdCho | - |  |  | **93.77** | *[91.97-94.75]* | **94.07** | *[92.10-95.44]* | *0.965* |
| Total PlsCho | - |  |  | **5.29** | *[4.01-6.28]* | **3.97** | *[3.49-6.33]* | *0.965* |
| Total PtdCho+PlsCho | - |  |  | **98.91** | *[98.67-99.01]* | **98.74** | *[98.40-98.97]* | *0.965* |
| PtdEtn16:0/16:1 | 688.49220 | 688.49118 | 1.49 | **1.56** | *[1.10-2.07]* | **1.20** | *[1.02-1.73]* | *0965* |
| PtdEtn16:0/16:0 | 690.50693 | 690.50683 | 0.14 | **1.20** | *[0.71-3.71]* | **1.15** | *[0.68-3.62]* | *0965* |
| PtdEtn16:1/18:2 + PtdEtn16:2/18:1 | 700.52864 | 700.52757 | 1.54 | **0.01** | *[<0.01-1.07]* | **0.08** | *[<0.01-1.33]* | *0965* |
| PlsEtn16:0/18:1 | 712.49319 | 712.49118 | 2.82 | **0.16** | *[0.13-0.19]* | **0.15** | *[0.13-0.21]* | *0.965* |
| PtdEtn16:0/18:2 + PtdEtn16:1/18:1 | 714.50912 | 714.50683 | 3.20 | **3.17** | *[2.59-3.52]* | **3.26** | *[2.35-3.95]* | *0.965* |
| PtdEtn16:0/18:1 | 716.52340 | 716.52248 | 1.28 | **17.62** | *[15.01-25.92]* | **19.56** | *[15.09-28.22]* | *0.965* |
| PlsEtn16:0/20:4 | 722.51344 | 722.51192 | 2.11 | **<0.01** | *[<0.01-1.41]* | **<0.01** | *[<0.01-2.29]* | *0.965* |
| unknown | 726.54467 | 726.54322 | 2.00 | **<0.01** | *[<0.01-0.70]* | **<0.01** | *[<0.01-0.72]* | *0.965* |
| unknown | 736.49412 | 736.49118 | 3.99 | **0.47** | *[0.05-0.57]* | **0.43** | *[<0.01-0.56]* | *0.965* |
| PtdEtn16:0/20:4 + PtdEtn16:1/20:3 | 738.50902 | 738.50683 | 2.97 | **14.97** | *[9.77-20.14]* | **12.91** | *[3.43-19.87]* | *0.965* |
| PtdEtn18:1/18:2 + PtdEtn16:0/20:3 | 740.52111 | 740.52248 | -1.86 | **4.50** | *[3.25-5.68]* | **3.38** | *[2.66-4.60]* | *0.965* |
| PtdEtn18:1/18:1 + PtdEtn18:0/18:2 + PtdEtn16:0/20:2 | 742.53745 | 742.53813 | -0.92 | **3.28** | *[2.42-3.93]* | **3.10** | *[2.52-4.34]* | *0.965* |
| PtdEtn18:0/18:1 + PtdEtn16:0/20:1 | 744.55291 | 744.55378 | -1.17 | **3.30** | *[2.93-5.07]* | **3.42** | *[2.57-4.79]* | *0.965* |
| PlsEtn16:0/22:6 | 746.51480 | 746.51192 | 3.87 | **<0.01** | *[<0.01-0.65]* | **<0.01** | *[<0.01-0.94]* | *0.965* |
| PlsEtn18:1/20:4 | 748.52925 | 748.52757 | 2.25 | **<0.01** | *[<0.01-2.41]* | **<0.01** | *[<0.01-3.31]* | *0.965* |
| PlsEtn18:0/20:4 + PlsEtn16:0/22:4 | 750.54332 | 750.54322 | 0.14 | **<0.01** | *[<0.01-5.51]* | **<0.01** | *[<0.01-8.38]* | *0.965* |
| PtdEtn16:0/22:6 + PtdEtn18:1/20:5 + PtdEtn18:2/20:4 | 762.50873 | 762.50683 | 2.49 | **5.10** | *[1.54-5.77]* | **3.99** | *[0.37-5.71]* | *0.965* |
| PtdEtn18:1/20:4 + PtdEtn16:0/22:5 | 764.52303 | 764.52248 | 0.72 | **6.19** | *[1.01-7.63]* | **5.30** | *[0.81-7.30]* | *0.965* |
| PtdEtn18:0/20:4 + PtdEtn16:0/22:4 | 766.53697 | 766.53813 | -1.52 | **10.01** | *[4.97-14.08]* | **9.04** | *[2.07-13.83]* | *0.965* |
| PtdEtn18:0/20:2 + PtdEtn18:1/20:1 + PtdEtn18:2/20:0 | 770.56637 | 770.56943 | -3.97 | **<0.01** | *[<0.01-0.19]* | **<0.01** | *[<0.01-0.16]* | *0.965* |
| PlsEtn18:1/22:6 | 772.52874 | 772.52757 | 1.52 | **<0.01** | *[<0.01-0.67]* | **<0.01** | *[<0.01-1.03]* | *0.965* |
| PlsEtn18:0/22:6 + PlsEtn18:1/22:5 | 774.54339 | 774.54322 | 0.22 | **<0.01** | *[<0.01-1.93]* | **<0.01** | *[<0.01-3.01]* | *0.965* |
| PlsEtn18:1/22:4 | 776.55758 | 776.55887 | -1.65 | **0.38** | *[<0.01-1.43]* | **0.29** | *[<0.01-1.71]* | *0.965* |
| PlsEtn18:0/22:4 | 778.57394 | 778.57452 | -0.74 | **0.33** | *[<0.01-1.92]* | **0.29** | *[<0.01-2.32]* | *0.965* |
| PtdEtn20:4/20:4 + PtdEtn18:2/22:6 | 786.50701 | 786.50683 | 0.23 | **0.84** | *[0.36.1.41]* | **0.76** | *[0.19-1.20]* | *0.965* |
| PtdEtn18:1/22:6 + PtdEtn20:3/20:4 | 788.52233 | 788.52248 | -0.19 | **1.44** | *[<0.01-2.56]* | **1.00** | *[<0.01-2.10]* | *0.965* |
| PtdEtn18:0/22:6 | 790.53652 | 790.53813 | -2.04 | **1.50** | *[<0.01-2.23]* | **1.16** | *[<0.01-1.89]* | *0.965* |
| PtdEtn18:0/22:5 + PtdEtn18:1/22:4 | 792.55218 | 792.55378 | -2.03 | **0.96** | *[<0.01-1.33]* | **0.82** | *[<0.01-1.26]* | *0.965* |
| PtdEtn18:0/22:4 + PtdEtn20:0/20:4 | 794.56836 | 794.56943 | -1.35 | **0.83** | *[0.20-1.00]* | **0.76** | *[0.05-0.99]* | *0.965* |
| PtdEtn20:0/20:2 | 798.60008 | 798.60073 | -0.82 | **<0.01** | *[<0.01-<0.01]* | **<0.01** | *[<0.01-<0.01]* | *0.965* |
| PtdEtn20:4/22:6 | 810.50746 | 810.50683 | 0.77 | **0.56** | *[0.31-0.79]* | **0.39** | *[0.17-0.67]* | *0.965* |
| PtdEtn22:6/22:6 | 834.50674 | 834.50683 | -0.11 | **0.26** | *[0.07-0.66]* | **0.26** | *[0.06-1.15]* | *0.965* |
| Total PtdEtn | - |  |  | **98.44** | *[83.16-98.90]* | **98.48** | *[75.38-98.86]* | *0.965* |
| Total PlsEtn | - |  |  | **0.97** | *[0.61-16.10]* | **0.87** | *[0.47-23.69]* | *0.965* |
| Total PtdEtn+PlsEtn | - |  |  | **99.37** | *[98.84-99.44]* | **99.40** | *[99.07-99.51]* | *0.965* |

*^a^*: [M+H]+ for PtdCho and PlsCho, and [M-H]- for PtdEtn and PlsEtn species

*^b^*: IQR: interquartile range

PtdCho: phosphatidylcholine; PlsCho: plasmenylcholine; PtdEtn: phosphatidylethanolamine; PlsEtn: plasmenylethanolamine

Abbreviations of individual PtdCho, PlsCho, PtdEtn, and PlsEtn species are as follows: position on the glycerol backbone as shown as *sn*-1/*sn*-2 of the fatty acid and fatty alcohol radicals (abbreviated as number of carbons: number of double bonds).
